# Verbal counting and the timing of number acquisition in an indigenous Amazonian group

**DOI:** 10.1371/journal.pone.0270739

**Published:** 2022-08-01

**Authors:** Isabelle Boni, Julian Jara-Ettinger, Sophie Sackstein, Steven T. Piantadosi

**Affiliations:** 1 Department of Psychology, University of California Berkeley, Berkeley, CA, United States of America; 2 Department of Psychology, Yale University, New Haven, CT, United States of America; 3 Department of Brain and Cognitive Sciences, University of Rochester, Rochester, NY, United States of America; University of Birmingham, UNITED KINGDOM

## Abstract

Children in industrialized cultures typically succeed on Give-N, a test of counting ability, by age 4. On the other hand, counting appears to be learned much later in the Tsimane’, an indigenous group in the Bolivian Amazon. This study tests three hypotheses for what may cause this difference in timing: (a) Tsimane’ children may be shy in providing behavioral responses to number tasks, (b) Tsimane’ children may not memorize the verbal list of number words early in acquisition, and/or (c) home environments may not support mathematical learning in the same way as in US samples, leading Tsimane’ children to primarily acquire mathematics through formalized schooling. Our results suggest that most of our subjects are not inhibited by shyness in responding to experimental tasks. We also find that Tsimane’ children (N = 100, ages 4-11) learn the verbal list later than US children, but even upon acquiring this list, still take time to pass Give-N tasks. We find that performance in counting varies across tasks and is related to formal schooling. These results highlight the importance of formal education, including instruction in the count list, in learning the meanings of the number words.

## Introduction

When children are first learning how to count, they begin by learning to recite the list of count words “one”, “two”, “three”, etc. without knowledge of their meaning [1, 2]. Children then progress through a stereotypical sequence of stages, known as knower-levels, where the meanings of each word are learned in succession [1, 3, 4]. A classical measure of knower-level is the Give-N task, in which subjects show their number knowledge by selecting different numbers of objects from a set [3]. When children are starting to learn the numbers, they initially learn the meaning of “one”, subsequently learn the meaning of “two”, and so forth. Some time after the “three” or “four” knower stage, children become *Full-Counters*, at which point they are able to use counting to determine exact cardinality. This knower-level pattern has been observed around the world (for an overview, see [5]). However, the timing of when children start progressing through these stages, and ultimately become Full-Counters, differs by language and culture [5–8].

For example, compare children in the US to children in the Tsimane’, a farming-foraging group indigenous to the Bolivian Amazon. Many Tsimane’ do not prioritize formal education compared to industrialized populations, and their engagement in economic activities requiring numerical knowledge is limited (an extensive description of the culture can be found in [9]). Prior work has documented differences in their learning trajectories for number words: the average US child learns the meaning of the word “one” (becoming a “1-knower”) around age 2 or 3; [8] found that the average Tsimane’ child is a 1-knower starting around age 5 or 6. While it takes the average US child on the order of 1–2 years to change from 1-knower to Full-Counter, the typical Tsimane’ child makes this transition around 4–8 years [8].

There are several possible reasons for Tsimane’ children’s differing trajectory. First, shyness might mask Tsimane’ children’s performance on number tasks. Studies suggest that willingness to communicate can be affected by various environmental factors [10, 11], including the presence of people from other cultures [12]. Children in Tsimane’ communities interact only occasionally with outsiders and even though the translators used are from the communities, it is possible that children feel shy talking in the presence of researchers. Our second hypothesis explaining Tsimane’ children’s number knowledge trajectory is that they do not memorize the verbal counting routine, “one”, “two”, “three”, …, before they start learning the number word meanings in the same way that US children do [1, 2]. [8] reported that 34% of a small sample of subset-knowers did not count out loud, and 24% counted to only four or less—potentially due to shyness, simply not knowing the count list words, or both. This list of initially “meaningless” words has been argued to provide a key structure for figuring out what these words mean and how their meanings interrelate [1, 13]. Thirdly, in contrast to many US children, Tsimane’ children may receive less general number input at home from their parents (e.g. cardinality labeling of sets of visible objects). This would lead to slower acquisition, just as differential parental input does in US samples [14, 15]. This possibility is independently supported by [16] who reported that Tsimane’ parents spend less than 1 minute per daylight hour of time speaking to their children, roughly 1/6th that of working class families in Boston. Instead, after age 3 children’s peers provide most of their linguistic input. To the extent that formal schooling is pursued, it could compensate for lower home number input. These hypotheses should not be thought of as mutually exclusive. Rather, they represent different levels of questioning to understand Tsimane’ children’s number development. The first hypothesis explores a methodological concern that might mask children’s knowledge, the second addresses a specific skill that might be responsible for the difference in timing, and the other pertains to the influence of context.

Which of these possibilities is correct—and more than one of them may be relevant—may have important implications for education. For one, if shyness plays a role in children’s responses, this can push us to improve evaluative practices in culturally appropriate ways. On the mathematical front, if the timing of number acquisition in indigenous communities happens to be primarily determined by knowledge of the verbal counting routine, that suggests a different focus for interventions than if the timing is more generally determined by formalized schooling (as opposed to home input). Mathematical literacy has been shown to be important for market and non-market outcomes (e.g. wealth, reported perceived stress, child health) in the Tsimane’ [17]. More generally, the determinants of number acquisition are important to understand for formulating and evaluating developmental theories. Nearly all theories of early number acquisition [1, 13, 18–20] call on more primitive, innate systems of representation [21] as the foundation of number learning even though these theories were primarily, if not exclusively, motivated by work on WEIRD samples [22] of learners. Children in the US and other industrialized cultures are in an unusual educational situation, with parents that are highly educated, care deeply about teaching number, and provide educational toys designed to practice counting. In contrast, work on non-WEIRD samples can inform how the mechanisms of conceptual change—or learning—and input operate more generally and are different between human groups.

We conducted a series of experiments with Tsimane’ children: a warm-up task, a verbal count list elicitation, a count list elicitation using tokens (“Move-and-Say”), and Give-N. We also collected demographic information including amount of formal education in the school classrooms present in each village. This data helped us arbitrate among hypotheses that could explain Tsimane’ children’s mathematics trajectory. Because shyness may lead children to not want to count out loud, our warm-up task is meant to to quantify and alleviate shyness in verbal behavior. Indeed our results show that most Tsimane’ children were willing to respond verbally in our tasks, largely eliminating shyness as a potential concern and allowing us to focus on characterizing their numerical knowledge. We were also interested in quantifying Tsimane’ children’s count list knowledge compared to their number concept knowledge (measured by Give-N). If their number performance in Give-N tasks is upper-bounded by the number *words* they know, we would find that these children do not verbally list count words higher than their Give-N level. On the other hand, we might find that children *are* able to verbally count higher than their Give-N level—regardless of the timing of acquisition relative to US children—and this would suggest that knowledge of the verbal list is not bounding their number concept acquisition. Data on years of formal schooling can help us determine whether input at home induces the particular mathematical skills we test, or if formal schooling is the determining factor in acquisition. In our research, we find that children know the count list beyond their Give-N level around the time they would be transitioning to Full-Counters, and that formal schooling is crucial to their numerical knowledge.

Finally, besides these primary measures, we administered variants of number tasks such as Give-N. Different versions of tasks can tap into slightly different components of children’s numerical knowledge. For instance, a Give-N task administered in count list order may have different demands than a Give-N task administered in random order. Likewise, a purely verbal task might have a different degree of difficulty than a verbal task accompanied by physical tokens. Proficiency can also be dependent on context, such as prior exposure to similar tasks. To establish a foundation for these sorts of speculations, we explored task differences in our research. We found that, in contrast to some US work reporting that children’s knowledge is robust across tasks [3], Tsimane’ children’s performance is dependent on the specific task.

## Materials and methods

The research was conducted in the summer of 2018, in various villages near the city of San Borja, Bolivia. We worked with a local research center, the *Centro Boliviano de Investigacion y de Desarrollo Socio Integral* (CBIDSI), working with members T. Huanca and R. Godoy. CBIDSI coordinated logistics, recruited participants, provided us with native translators, and served as experts on Tsimane’ culture. Our studies were approved by the Gran Consejo Tsimane’ (Tsimane’ Grand Council) and by our institutional IRB. Experimental studies were conducted in each village’s schoolhouse. Demographic information was obtained by surveying the parents of younger children, and obtaining the self-report of older children. Formal education was measured in number of years parents reported that children had attended school, although we note that these numbers are not generally comparable across cultures due to different educational practices and variability in school attendance.

### Participants

We recruited 109 Tsimane’ children, ages 4–11 (43 female, 57 male), but excluded 9 due to fussiness, clear misunderstanding, disinterest in the task, or excessive parental interference. These 9 subjects were excluded in the data collection phase, and were never included in any analysis, visualization, or in any other way throughout the study.

After data collection we had 100 subjects, on whom we conducted warm-up task analyses. Among these 100 children, we identified 11 shy children. These children were excluded from the analyses for the tasks following the warm-up task, leaving 89 children in the remaining analyses.

### Procedure

**Fig 1:**
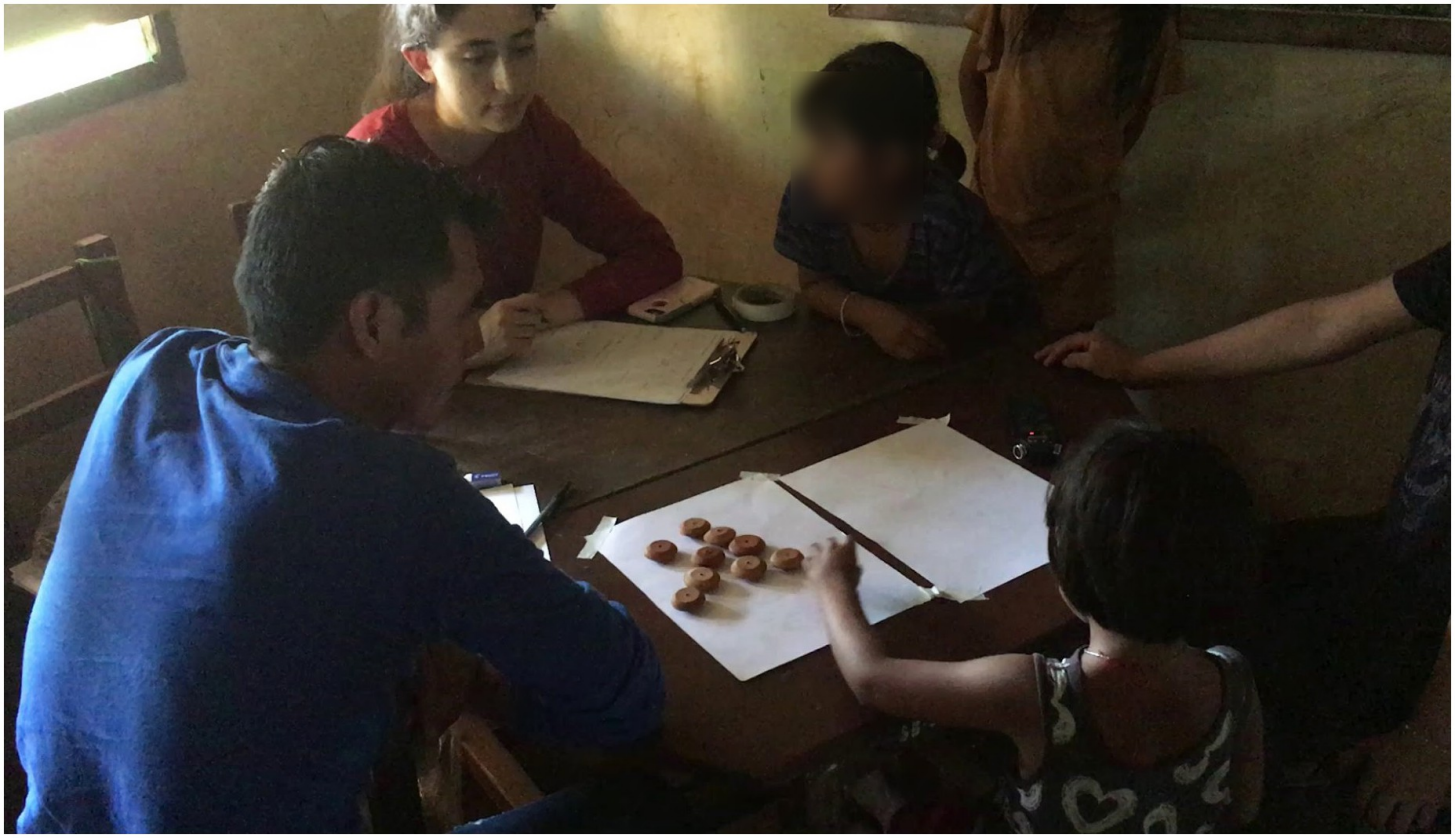
Setup. The basic setup for all tasks: two pieces of white paper that children are asked to move tokens between. In the photograph, Give-N is being administered. Note that it is generally difficult to separate individuals for private testing.

Children were tested in four primary tasksin the following order: (i) a warm-up listing task unrelated to counting that was designed to detect and/or alleviate shyness, (ii) elicitations of the verbal count routine in Spanish and Tsimane’, (iii) a “Move-and-Say” task, and (iv-v) different Give-N tasks. To complete these, the participating child was seated at a table next to a translator, as illustrated in Fig 1. All communications with the participant were in Tsimane’ (besides number in select cases— see below). Both child and translator were across from the experimenter. Two pieces of white paper, used as targets for the Give-N task, were placed next to each-other on the table, separated by a ∼1-inch gap. We discuss the details of each task in turn:

(i) **Warm-up task**: Children were randomly divided into two conditions. In condition A, the participating child was asked to name as many friends as possible, and then as many vegetables as possible. “Some of my friends are [3 examples]. Can you name some friends?” “Can you name some vegetables? Like: tomato, potato, onion…” In condition B, the child was asked to name as many body parts as possible, and then as many letters of the alphabet as possible. “Can you name some body parts? Like: nose, stomach, arm…” “Can you name some letters of the alphabet? Like: A, B, C….” If the child paused, naming more items was encouraged, until the child could no longer name any items. Based on pilot data collected on a previous trip to Bolivia (not included in the data reported here), we expected that the elicited lists would vary in difficulty; i.e. with children able to name more friends and vegetables than body parts and ABCs. Our pilot data also suggested that harder lists might lead to decreased number listing, effectively providing a manipulation of shyness or confidence in the mathematical tasks. (We ultimately abandoned this idea of employing list difficulty as a shyness manipulation, because it did not prove to be effective in our larger sample, as explained further below).
  Warm-up task identity aside, quantity of items named allows us to quantify shyness: for simplicity we use a binary categorization where we consider individuals to be *shy* if they do not name any warm-up items (from either of the lists) beyond the baseline number of items provided to them by the experimenter, and *not shy* if they name items above the baseline provided. As previously mentioned, we remove these shy children (n = 11, leaving 89 children) from our analyses of the tasks we performed after the warm-up task.
  One caveat is that these children might be uneducated rather than shy, or have a lower education *and* be shy. In spite of this ambiguity, we considered it more important to exclude potentially shy children who might have performed differently on verbal and non-verbal tasks merely because of social disposition, rather than understanding. (Also, in spite of the possibility that the children we exclude are disproportionately lower-education, we still obtain a varied representation of educational levels after this exclusion).
(ii) **List Routine Elicitation**: Participants were asked to recite the Spanish count routine first, then the Tsimane’ count routine. In both cases, the child was guided by the experimenter with the phrase “Can you count with me? 1,2,3 …” on the first three items. This means that children who fail to know verbal labels for less than “four” would not be detectable with this task, but we decided in designing the task that it was more important that children have a little guidance in order to feel secure in counting in order to minimize effects of shyness.
(iii) **Move-and-Say**: For Move-and-Say count list elicitation, twenty plastic chips were placed on the paper on the child’s left. The researcher asked, “Can you count with me? 1,2,3….”, transferring a chip to the paper on the right with each recited number. As the child continued the count list, the researcher continued transferring a chip each time the child named an item. The language for the numbers in Move-and-Say was determined by the experimenter’s real time judgment for which language the child had been more proficient in in the list routine elicitations. In cases where the child was deemed equally proficient, the child was explicitly asked in which language they wished to hear/say the numbers. This language of higher proficiency was also used for all subsequent tasks. (Note that while only 4% of children spoke Spanish “well”–as opposed to “a little” or “not at all”– 59% of children were better at naming the numbers in Spanish. We believe this is because their school taught them the numbers in Spanish).
(iv) **Ordered Give-N**: We used simplified Give-N tasks with circular wooden tokens, of 1.5’’ diameter. We asked children to move between 1 and 8 tokens from the left piece of paper to the right, and the pile from which they drew tokens always contained 10 tokens (in order to detect giving all as a response). The child was asked: “Can you move N to this other paper?”. In the first Give-N task, ordered Give-N, the number of tokens the researcher asked the child to move increased in order. First the child was asked to move 1 token, then they were asked to move 2 tokens, and so on, until they were asked to move 8 tokens. This task was run only once. Important differences to prior tasks (e.g. as in [8]) include that children were *not* asked to re-count or check their answer at the end. This ensured that they could complete the task quickly, easily, and with minimal social or pragmatic pressures. In addition, this means that performance on the task is determined only by knowledge of Give-N, and not by abilities to answer How-Many or Are-There-N.
(v) **Random Give-N**: In the final task, we gave children the Give-N task in random order based on a random number generator. Because every presentation of Random Give-N is in a new random order, we administered the task two consecutive times per participant, to determine if different random orders of presentation would affect performance. We found no major differences in performance between the first and second administration of random Give-N (r = 0.87), so we base all our analyses of random Give-N performance on the first administration. Moreover (except when we explicitly compare random Give-N to ordered Give-N performance towards the end of our results), we base all our analyses of Give-N performance on the first administration of random Give-N. (As we explain more at length further on, it is difficult to know what version of a number task will be a valid measure of an underlying cognitive capacity. We select random Give-N as a representative of Giver-Level on the assumption that the random version requires a more robust ability to count than ordered Give-N. Refer to the discussion for further detail on task differences).

The total amount of time testing took per child was approximately 15 minutes. Participants were compensated with educational materials (pens, pads, and erasers) and culturally-appropriate local goods (toothpaste, toothbrush, soap, juice, cookie).

### Scoring children’s performance

We aimed to put this data into a uniform format where tasks could be compared on the same footing. To do this, we coded each child’s performance on a task as the highest number they got correct until they made a mistake. For example, if a participant verbally counted “one,”, “two,” “three,” “four,” “six,” “seven,” we considered that participant to be a *4-Lister*. We calculated Lister-Level for the Spanish and Tsimane’ count routine tasks separately, and then for each subject, determined their best performance out of the two elicitations. This best performance is what “Lister-Level” refers to throughout our analysis. This step helps to ensure we are measuring a child’s actual listing capabilities, in whichever language they are most comfortable counting. (If they performed equally—for example listing to 9 in Spanish and Tsimane’, their Lister-level was simply recorded as that common number: 9, in this case).

We also computed a “Move-and-Say Level” and “Giver-Levels” for random and ordered Give-N, again based on the highest number they reached without making a mistake. Level was computed in the random order Give-N by numerical order, not order in the task. We use the term “Subset-Givers” to refer to children who are non-Givers, 1-Givers, 2-Givers, or 3-Givers. We call levels of children’s performance “Giver-level” rather than “Knower-level” because of the relatively simple way we compute it (compared to e.g. [4, 23–25]). On the downside, this scoring scheme ignores some of the complexities of learning; for instance, that children often learn an incorrect but stable verbal counting routine [2]. However, trying to model irregularities in comparisons would require much longer tasks that were inappropriate with our population, and cause difficulties in comparing across tasks. (As a side-note, with our methods, we occasionally we find 5, 6, and 7-givers, but we are not making a theoretical claim about distinct developmental stages straying from classical knower-levels; our simplified method of classification is simply meant to describe a general pattern of Give-N competence progression in the data).

Field work often raises unique challenges in coding data. Due to the population’s interest in our tasks and the general inability to isolate subjects during testing, onlookers often crowded around to watch the individual participating. On occasional trials an observer would shout out the answer. In these cases, we consider the participant to be accurate if they were correct on the next item in the task. For example, in verbal count list elicitation, if the subject recited up to 9, an observer shouted out “10”, and the subject continued listing starting at 11, we would count 10 as if the subject had said it. On the other hand, if the subject listed up to 9, an onlooker shouted “10”, and the participant next said “12”, we would say the participant listed up to 9. If an onlooker interfered with an item on the Give-N task, we asked the participant for the target quantity again at the end of testing, and recorded the participant’s performance on that repeated trial as the participant’s response. If an onlooker interfered in a warm-up task, we did not include the answer shouted out because the space of possible warm-up answers is larger, and it seemed unlikely that the child would have come up with that specific answer (e.g. that specific vegetable that was shouted out). The exception is the ABC task, where we implemented the same procedure as in the verbal counting tasks of only counting the participant’s answer as correct if they were correct in the next letter in the alphabet.

To simplify our analyses, and because we are only interested in the range of numbers that are learned when children are first learning how to count, we cut the children off at 20 items listed. For consistency across different tasks, we also cap warm-up task items listed at 20.

## Results

We break down our analysis by the primary questions of theoretical importance.

### Tsimane’ children are generally not too shy to respond in verbal tasks

**Fig 2:**
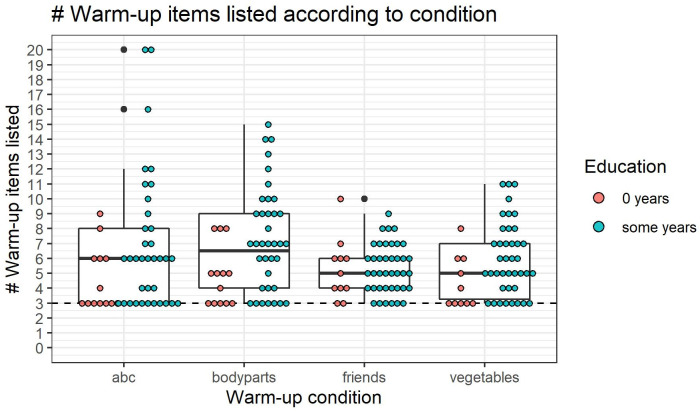
Warm-up items listed per warm-up condition. How many warm-up items were listed for each warm-up condition. As in the other tasks (except for Give-N), the baseline number of items named is set to y = 3 for every child, because children were given 3 example items to verbalize before being asked to continue listing items on their own. For easier visualization, children’s count list responses have been cut off at 20. Individual dots are color-coded by education.

We first examined behavior in the warm-up task (i). Fig 2 shows the number of items listed (y-axis) for each warm-up category (x-axis). Each point represents an individual child and color shows whether or not the child has attended school. Despite the unusualness of researcher visits to Tsimane’ villages and the unfamiliarity of testing situations, Tsimane’ children *were* willing to provide answers to the requested lists. Most children (binomial proportion test *θ* = 89%, *p* < 0.001, 95% CI: [0.81% to 0.94%]) listed above warm-up baseline, indicating that shyness was generally not a barrier to verbalizing knowledge. In fact, generally, Tsimane’ children were willing to name around three more items than the experimenters named in each list (i.e. the medians in Fig 2 are about 6, or 3 more than baseline).

We note that the two conditions (ABCs/body parts vs. friends/vegetables) did not reliably differ in the average number of items that children were willing to provide, contrary to our initial speculation that children would be more willing to list friends and vegetables as opposed to ABCs and body parts (*p* = 0.25, Wilcox test *W* = 1082.5; we analyze this data using a non-parametric test of means due to the non-normal distribution of responses). This suggests that our participants did not actually consider one task more difficult than the other, which means the warm-up tasks should not be treated as distinct manipulations of shyness. Observing number of items named in the warm-up tasks, however, is still informative as a measure of shyness.

In both 0/some years education categories, the minority of children were at warm-up task baseline. (0-years education prop.test: proportion = 0.065, *p* < 0.01, 95% CI: [0.02 − 0.15]. Some-years education prop.test: proportion = 0.26, *p* < 0.05, 95% CI: [0.11 − 0.49]). This shows that naming warm-up items is not exclusively reserved to children with formal education. At the same time, these results also show that education is likely to be a factor in responding. The overall median number of responses for children who reported having some education was 12 and for no education was 9, a difference which was statistically reliable using a Wilcox test (*p* < 0.01, Wilcox test *W* = 1209). Also, there appears to be a weak positive correlation between number of warm-up items named and education (r = 0.37, p < 0.01).

### Warm-up task does not affect Tsimane’ number task performance

**Fig 3:**
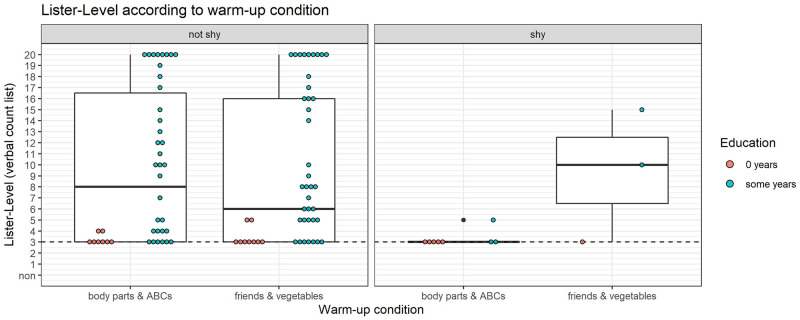
Count list items listed per warm-up condition. How many items of the verbal count list were named under each different warm-up condition. The plot is divided into two panels, the rightmost representing children who are shy (the minority). Children with zero years reported formal education (red dots) have lower Lister-Levels than children with some formal education (teal dots).

Consistent with the fact that children named a statistically equal number of items in both warm-up tasks, type of warm-up task children were assigned to had no effect in how many items children named in our number tasks. To illustrate this, we focus primarily on the numerical listing task, as it immediately succeeded the warm-up task and could directly test if there was an immediate effect of warm-up without interference from other tasks. Fig 3 shows the number of verbal count routine items in the count routine task (ii) that children produced (y-axis) as a function of their non-numerical list in the warm-up task (i) (x-axis). Across all children, 66% percent have Lister-Levels above baseline and typically list around 6–8 items. The different warm-up conditions appeared not to affect later number word production in the Tsimane’ (*p* = 0.38, *W* = 1374.5). These results are in contrast to US controls we tested, who *were* biased by warm-up task (*p* < 0.01, W = 396). (See S1 Appendix for further details).

### Education is associated with higher number list performance

Fig 3 also shows that children with more formal education (teal) tend to name more count list items than children with *no* education (red) (*p* < 0.01, Wilcox test *W* = 222.5). Only 17% of children with no education have Lister-Levels above baseline. While it is likely that this relationship reflects causal influences of education on counting, it is also possible that there is a selection bias where children with less mathematical knowledge—perhaps because their parents have fewer years of education themselves—tend not to attend school.

### When children don’t verbally count, it is mostly due to not knowing the words rather than shyness

Fig 3 also groups Tsimane’ children by their shyness level. Here, we see that the majority of children who fail to verbally count (i.e. are at a Lister-Level of 3) are *not* shy, meaning that they will list items above baseline that are not number words. Thus, children who performed at baseline in listing numbers probably genuinely do not know number words. Of children at baseline for naming count list items, 8 did not provide any warm-up items, but 26 did. This suggests that over 3/4 (76.47%; 95% CI: [58.43% to 88.62%]) of children who do *not* count are doing so, not out of shyness since they will name other words, but out of genuine lack of knowledge of the counting routine.

As seen in the right-hand panel of Fig 3, there are a few children who *are* categorized as shy according to the warm-up task. These children generally do not provide many verbal number words. As previously outlined, shy children (n = 11, leaving 89) were removed from all following analyses (n = 11 shy children were removed from 100 subjects, leaving 89 subjects for subsequent analyses).

### List knowledge is unlikely to be a bottleneck in becoming Full-Counters

Recall that Tsimane’ children’s progression through knower-level stages occurs later and takes more time than that of children from industrialized samples. Our first analysis examines children’s list knowledge around the time they become Full-Counters, defined as succeeding on 5 or beyond on the Give-N task. If not knowing the verbal routine explains why children take longer to transition to Full-Counter we should find that children’s Lister-Level *does not exceed* their Giver-Level; if the count list is not the bottleneck, we expect their Lister-Level to exceed their Giver-Level.

**Fig 4:**
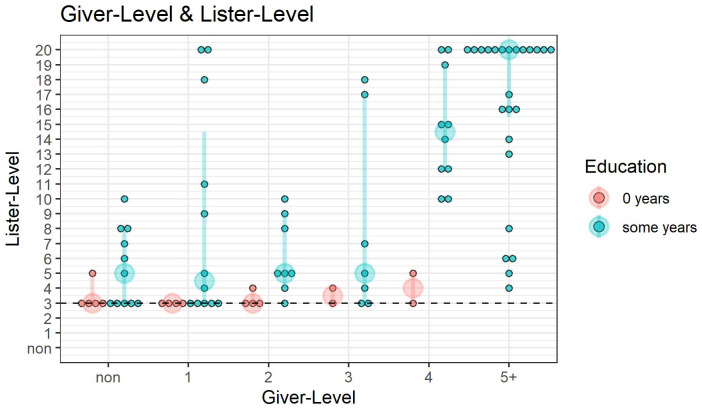
Lister-Level by Giver-Level. This shows that 4-Givers almost always have Lister-Levels above their Giver-Level, and non-, 1-, 2-, and 3-Givers mostly don’t do as well. Each small, black-bordered point represents an individual child. The larger, transparent red and teal dots represent the bootstrapped median Lister-Level corresponding to a particular Giver-Level and educational status (color-coded).

Fig 4 shows Lister-Level (y-axis) as a function of Giver-Level (x-axis). Bootstrapped median Lister-Levels for each Giver-Level are represented by the larger transparent dots (color-coded red and teal according to educational status) with 95% CI error bars. For 4-Givers (whose next step is to become Full-Counters), children’s Lister-Level is almost always higher than their Giver-Level. By the time children are succeeding on 4 on the Give-N task, they are able to list up to around 15 number words (with the exception of the two 4-Givers who have received no formal education). By the time they are succeeding on 5 and beyond on Give-N, they can typically name around 17 or more number words. This indicates that children do tend to know more words than they are able to use in the Give-N task, at these higher knower-levels.

We performed a regression to statistically evaluate the trends in Fig 4. The regression is dummy-coded, and treats the predictors of Giver-Level and Education (0/some years) as categorical factors. The results of this regression in Table 1 show that 4-Givers reliably listed on average 7.5 items above baseline (*β* = 7.50, *t* = 4.06, *p* < 0.001) and 5+-Givers reliably listed on average 9.71 items above baseline (*β* = 9.71, *t* = 6.02, *p* < 0.001). Besides this, across all giver-levels, children with “some” education reliably list about 4.54 more numbers than children with no education (*β* = 4.54, *t* = 3.31, *p* < 0.01). The adjusted R-squared (0.49) indicates that about half of the variance in Lister-level is explained by Giver-level and Education.

**Table 1:** Output for regression: Lister-Level ∼ Giver-Level + Education. The predictors are both dummy-coded and categorical. Education is a binary predictor with two levels: “0 years” and “some years”.

	Estimate	SE	95% CI lower	95% CI upper	t	p
Intercept	1.63	1.53	-1.41	4.67	1.06	0.29
Giver-level 1	2.09	1.70	-1.30	5.48	1.23	0.22
Giver-level 2	0.51	1.84	-3.15	4.17	0.28	0.78
Giver-level 3	1.95	2.01	-2.05	5.95	0.97	0.33
Giver-level 4	7.50	1.85	3.83	11.18	4.06	0.00
Giver-level 5+	9.71	1.61	6.50	12.91	6.02	0.00
Education (some)	4.54	1.37	1.81	7.27	3.31	0.00

In summary, this part of Tsimane’ children’s number learning trajectory (4 and 5+-Giver status) mirrors US populations where verbal behavior exceeds the ability to use numbers in a task like Give-N. This suggests that lack of knowledge of the Lister-Level is not preventing children from becoming Full-Counters (i.e. 5+-Givers).

### Verbal knowledge may constrain Subset-Givers

Fig 4 also shows that non-, 1-, 2-, and 3-Givers typically do *not* list many number words. Recall that to help children understand and feel comfortable, our task gave three examples of number words (“Can you count with me? 1, 2, 3 …”). This means that for median Lister-Levels we report as 3 on Fig 4, it is ambiguous whether the subject’s true Lister-Level is 3 or below. Therefore, we cannot tell whether or not these participants consistently have a higher Lister-Level than their Giver-Level.

Our earlier regression can again corroborate the qualitative observations from Fig 4. In Table 1, note that non-Givers (*β* = 1.63, *t* = 1.06, *p* = 0.29) do not list numbers reliably above the baseline of y = 3 list items. The Lister-levels of 1 (*β* = 2.09, *t* = 1.23, *p* = 0.22), 2 (*β* = 0.51, *t* = 0.28, *p* = 0.78), and 3-Givers (*β* = 1.95, *t* = 0.97, *p* = 0.33) trended 0.51 − 2.09 items higher than that of non-Givers, but the difference was not statistically reliable.

The fact that non- and 1-Givers typically do not list more than 3–5 number words contrasts with the trajectory observed in the US, where children are often able to learn up until ten or higher *before* they become one-knowers [2]. If knowledge of the list of number words [26]—independent of their meaning—is important to early learning, this might account for part of Tsimane’ children’s number acquisition timing, especially for those children with 0 years of education.

### Tsimane’ children start learning the count list and number meanings at a later age than US children

**Fig 5:**
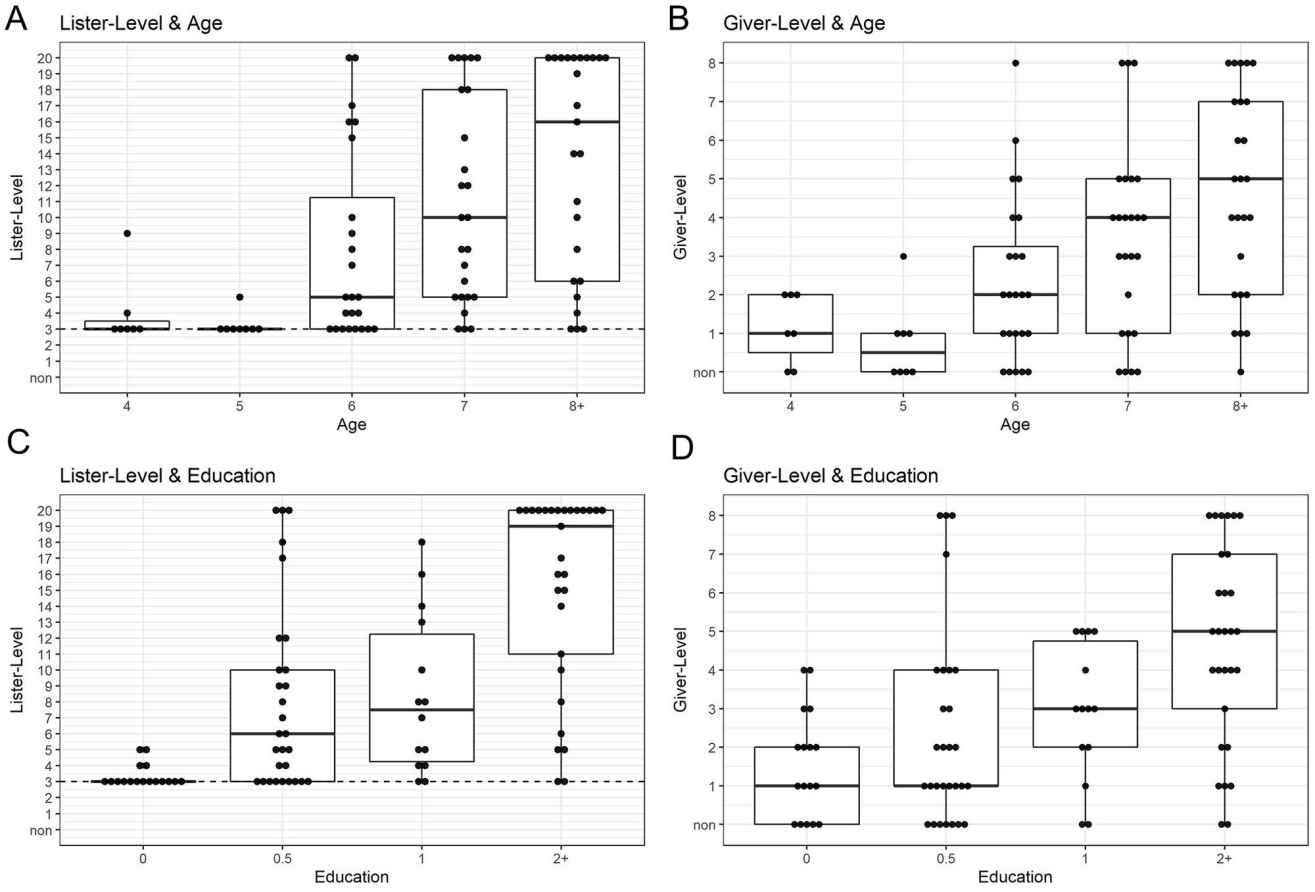
Lister and Giver-Levels by age and education. Increasing age and education are related to increasing Lister- and Giver- Levels. Because age and education are related, it is difficult to determine whether age, education, or both are independently driving Lister- and Giver- Level. However, it is likely that education is the primary catalyst for increasing Lister-and Giver- Level. (A) Lister-Level by age. Older children tend to have higher Lister-Levels than younger children. (B) Giver-Level by age. Older children tend to have higher Giver-Levels than younger children. (C) Lister-Level by education. Recall that children typically enter school at around 6–7 years old. Children with more formal education tend to have higher Lister-Levels. (D) Giver-Level by education. Children with more education tend to have higher Giver-Levels.

In order to visualize the relationship between education, age, and performance on these tasks, Fig 5 shows Lister- and Giver- Levels broken down by age and education. (The number of participants in our sample per age group are: 4 yrs: 7, 5 yrs: 8, 6 yrs: 24, 7 yrs: 25, 8–11 yrs: 25. The number of participants per educational group are: 0 yrs: 17, 0.5 yrs: 29, 1 yrs: 14, 2 yrs +: 29).

Fig 5 Panel A, plotting Lister-Level as a function of age, shows that at ages 4 and 5, Tsimane’ children’s Lister-Levels are at 3 or below. For comparison, in the US, 4 and 5 year old children count verbally higher than even ten or twenty (and are typically already Full-Counters). It is not until Tsimane’ children are 7 or 8 years old that they name a substantial number of number words. Fig 5 Panel B, depicting Giver-Level vs. age, shows that Tsimane’ children are still early Subset-Givers at ages 4, 5, and 6, ages at which American children would already be Full-Counters, replicating the timing reported in [8]. Only around age 7 do Tsimane’ children become 4+-Givers. Fig 5 Panel C-D show similar trends with years of education, showing that children’s Lister-Level and Giver-Level are both also correlated with years of education. Interestingly, many of the children with 0 years of education are essentially at floor on the Lister-Level task, but succeed on at least several numbers on the Giver-Level task.

We performed two regressions to jointly quantify trends in Lister-Level and Giver-level from continuous, centered predictors: education, age, and their interaction. These, shown in Tables 2 and 3, reveal statistically reliable influences of age on both, likely education on both (although the p-value is marginal for Giver-Level), and no reliable interactions. One observation to note is that there are bigger effects of age and education on Lister-Level than on Giver-Level. This may be because at home, there is little practical use for reciting many elements of the count list, but there is usefulness in knowing low, subitizable numbers (e.g. to retrieve objects for parents). Another observation is that there superficially appears to be a larger effect of age than education in predicting both lister-level and giver-level. However, it is difficult to conclusively differentiate between the influence of age and education, as they are correlated. We discuss this more at length subsequently.

**Table 2:** Output for regression: Lister-Level ∼ Education + Age + Education*Age. The predictors are continuous and de-meaned.

	Estimate	SE	95% CI lower	95% CI upper	t	p
Intercept	9.64	0.63	8.40	10.89	15.36	0.00
Education	1.16	0.34	0.49	1.84	3.43	0.00
Age	1.83	0.50	0.84	2.82	3.67	0.00
Education*Age	-0.06	0.20	-0.45	0.33	-0.29	0.77

**Table 3:** Output for regression: Giver-Level ∼ Education + Age + Education*Age. The predictors are continuous and de-meaned.

	Estimate	SE	95% CI lower	95% CI upper	t	p
Intercept	3.00	0.24	2.51	3.48	12.24	0.00
Education	0.27	0.13	0.01	0.53	2.03	0.05
Age	0.80	0.19	0.41	1.18	4.10	0.00
Education*Age	0.04	0.08	-0.11	0.19	0.51	0.61

### Rather than at home, Tsimane’ children start learning the count list and number meanings when they enter school

The regressions in Tables 2 and 3 reveal a reliable increase in both Lister-level with education (*β* = 1.16, *t* = 3.43, *p* < 0.001), and Giver-level with education (*β* = 0.27, *t* = 2.03, *p* < 0.05). Complicating the analysis, even though Tsimane’ children enter school later than US children, education is still somewhat correlated with age (*τ* = 4.7, *p* < 0.001), making it difficult to differentiate effects of age and education based on these continuous predictor regressions alone. However, a regression (adjusted R-squared: 0.2253) of Lister-Level ∼ Age treating age as a categorical predictor (Table 4) adds more to the story. Age 7 appears to be a special age: it is the first age when we see a lister-level (on average 10.80) reliably above baseline (*β* = 6.80, *t* = 2.68, *p* < 0.01). A regression (adjusted R-squared: 0.2224) of Giver-Level ∼ Age treating age as a categorical predictor (Table 5) yields similar results: age 7 is the first age at which we see a giver-level (on average 3.4) reliably above baseline (*β* = 2.26, *t* = 2.36, *p* < 0.05). What happens around age 7 or 8 that bumps up Lister- and Giver-Levels? This shift happens to coincide with the typical age Tsimane’ children enter school. The mean age for children who reported no education is 5.3 years old, 6 months of education is 6.5 years old, and 6 or more months of education is 6.9 years old. This indicates that what happens that accelerates Tsimane’ learning around age 7 is schooling. This importance of the age of school entry suggests that Tsimane’ children are not receiving a sufficient amount or relevant type of numerical input at home before entering school. It also suggests that education is really the important factor, rather than age.

**Table 4:** Output for regression: Lister-Level ∼ Age. Age is a dummy-coded, categorical predictor.

	Estimate	SE	95% CI lower	95% CI upper	t	p
Intercept	4.00	2.24	-0.46	8.46	1.78	0.08
Age 5	-0.75	3.07	-6.86	5.36	-0.24	0.81
Age 6	3.88	2.55	-1.19	8.94	1.52	0.13
Age 7	6.80	2.54	1.76	11.84	2.68	0.01
Age 8+	9.56	2.54	4.52	14.60	3.77	0.00

**Table 5:** Output for regression: Giver-Level ∼ Age. Age is a dummy-coded, categorical predictor.

	Estimate	SE	95% CI lower	95% CI upper	t	p
Intercept	1.14	0.85	-0.54	2.83	1.35	0.18
Age 5	-0.39	1.16	-2.70	1.91	-0.34	0.74
Age 6	1.19	0.96	-0.72	3.10	1.24	0.22
Age 7	2.26	0.96	0.35	4.16	2.36	0.02
Age 8+	3.50	0.96	1.59	5.40	3.65	0.00

Fig 5 Panels C and D concretely illustrate the overall trend of higher educational levels corresponding to higher Lister- and Giver-Levels. In Fig 5 Panels C, children with no education are almost all at baseline Lister-Levels. At half a year of education, children’s median Lister-Level already increases to 6 number words, and at 2 or more years of education, several children can list to 19 and beyond. Correspondingly, in Fig 5 Panels D, children having between 0 and 1 years of education usually have Giver-Levels below 4, but note that many zero-years education children have Giver-Levels of one or higher, indicating that some input from parents or peers teaches these skills before school. Children with 2 or more years of education appear to be advancing to Full-Counter status. Overall, it seems clear that education is associated with differences in Lister-level and Giver-level.

### Performance differs across numerical tasks

**Fig 6:**
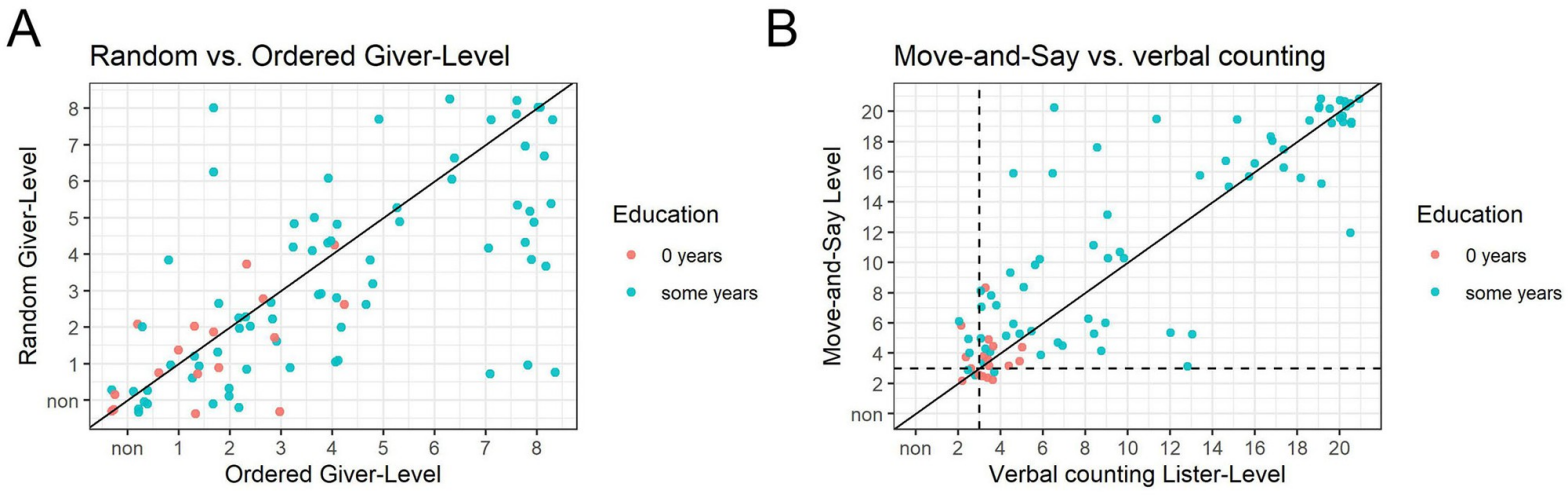
Task differences. Scatter plots showing performance of individuals on different tasks related to counting. Dots represent individual subjects and are jittered to prevent overlap. Participants are color-coded by education.

Finally, we were interested in examining the relationship between different components of children’s numerical knowledge. Fig 6A shows children’s relative performance on random and ordered Give-N. Dots below the line *y* = *x* represent children who perform better on the ordered task than on the random task, and dots above the line represent children who perform better on random than ordered Give-N. Specifically, 36 children perform better on ordered Give-N, 17 perform better on random Give-N, and 36 perform equally on both tasks. The majority (67.92% = 36/53) of children whose performance on ordered and random Give-N differs perform better on the ordered version than on the random version (prop. test *p* = 0.01, 95% CI: [0.54% to 0.80%]) (Fig 5A). Note that the children who perform most differently in these tasks tend to be ones at ceiling on ordered Give-N but below ceiling on Random Give-N (i.e. the right side of the plot), and all of these children have nonzero years education. This may suggest that some educational practices support Ordered Give-N over Random.

Fig 6B shows a comparison between Move-and-Say and verbal count list reciting. Most of participants who differ on these tasks perform better on Move-and-Say than on verbal listing (66%, prop. test *p* = 0.03, 95% CI: [0.51% to 0.78%]), even though both of these tasks are closely related. Specifically, 33 children perform better on Move-and-Say, 17 perform better on verbal listing, and 39 perform equally on both tasks. For children with no education (red dots in Fig 6B), there is no systematic difference in tasks, although this sample is small and children are near floor.

## Discussion

Our work charts out some of the details of how Tsimane’ children begin learning counting words and their meanings. These studies were motivated by previously observed differences in timing for their progression through knower-level stages relative to industrialized populations. Our results show that while Tsimane’ children learn the verbal counting list later than US children, by the time they are 4-Givers, they have learned enough of it that count list knowledge should not prevent them from reaching later Giver-Levels and becoming Full-Counters. In addition, Tsimane’ children are willing to provide verbal lists of various types, meaning that verbal elicitation is a viable method for them and other indigenous groups, despite the peculiarity of psychology research to these communities. This suggests that when Tsimane’ children do not provide verbal counting words, generally this is likely to be due to their particular underlying knowledge, not merely shyness.

We suspect that factors beyond the verbal list are largely responsible for the later and protracted timing of when Tsimane’ children become Full-Counters. One explanatory candidate is input, such as formal education or informal teachings from parents and peers. We found that in the Tsimane’, reported amount of formal education is an important factor in both verbal list knowledge and Giver-level. Tsimane’ children often do not attend school until they are 6 or 7 years old, at which point they start learning the verbal count routine, become Subset-Givers, and eventually become Full-Counters. The importance of this specific age, the age of school entry, suggests that although age and education are correlated, education is likely the key factor supporting number learning. Corroborating this idea that education is key, US children are able to progress through these stages at a younger age. An ongoing meta-analysis from a number of prior studies also highlights the importance of education; in an extended data set, *only* children with more than 0 years of education are found to be Full-Counters [27].

One detail to bear in mind is that even with formal schooling, Tsimane’ children take more time to progress through the stages than US children. This might be because Tsimane’ children attend school and/or study at home less frequently than US children do [28], perhaps tasked with culturally specific chores (e.g. as Mayan children are [29]). In parallel, there are reports of less parent-child verbal interaction in early childhood, with children receiving on average 1m of direct input from parents each daylight hour [16]. Thus, insofar as number words are being used at home, it may take longer for Tsimane’ children to amass the same amount of data about number words as US children. Additionally, outside of classrooms, teaching in small-scale societies tends to rely on observation and apprenticeship, rather than direct instruction [30, 31]. While these forms of teaching have been optimized for culturally relevant skills (e.g. fishing, gardening, hunting, and other foraging in the Tsimane’ [32]), this form of home teaching may not be as optimal for abstract abilities like number learning.

Our results highlight the importance of cultural support in advancing number learning. The centrality of cultural supports (e.g. parents, in US kids’ cases, or teachers, as it seems to be the case for Tsimane’ kids) in learning counting skills might also explain performance in different types of tasks. For example, Tsimane’ children may perform better in ordered Give-N than random Give-N if it is closer to skills explicitly taught in schools. Our findings are consistent with this account: when there were discrepancies in performance across different versions of tasks, they tended to occur in formally educated children. There are other examples of practice and context influencing numerical ability including studies of market vs. school arithmetic in Brazilian [33, 34] and Indian [35] children, and in anthropological analyses of the influences of economics and trade in numerical literacy [36, 37]. Beyond context, different tasks may have different demands on domain-general cognitive load, or working memory.

These task comparisons supply evidence counter to previous claims (based on US children) [3] that posit an easily acquired, readily generalizable, abstract conceptualization of number. This sort of theory would manifest in comparable performance across different counting-related tasks. Instead, understanding of counting is gradual: counting is a complex ability comprised of various sub-skills, and children appear to learn different components of counting at different times. These task differences also serve as a reminder to be careful and specific in interpreting cognitive measurements.

Our results also relate to the question of how important language may be for number concept acquisition. Some previous research has de-emphasized the importance of language. For instance, [38] administered exact number tasks that could be solved using language but do not require it. These tasks were given to indigenous children in the Australian Northern Territories whose languages (Warlpiri and Anindilyakwa) do not include high exact number words, and English-speaking children from Melbourne. Children’s performance was equal, irrespective of language group. NT children relied on spatial strategies to solve these tasks [39], which Melbourne children rarely used, relying on language instead. Other research emphasizes that language is helpful in supporting number. [40, 41] administered non-linguistic, exact number tasks to the Pirahã, an Amazonian group whose language has no exact number words. While subjects succeeded on several tasks, they had trouble in tasks involving larger sets and memorization. Here, language was not required for using numerical concepts, but would have been a beneficial cognitive tool to enhance performance. Relatedly, [42] showed that Tsimane’ participants’ exact number performance on non-linguistic matching tasks is bound by their verbal count range. In our present study, Lister-level almost always exceeded Giver-level. This is consistent with patterns observed in US children [2] that implicate the count list as a potential scaffold, or catalyst, for counting [1]. The fact that children’s verbal list knowledge preceded their Give-N number knowledge supports the notion that language is helpful in number understanding.

Overall, results from our particular population suggest that the home input provided to children by parents in indigenous communities in the absence of formal schooling is unlikely to be sufficient to learn large exact numbers (potentially explaining why communities like the Munduruku or Pirahã [41, 43] do not have exact number words). While we found formal education to be an important predictor of understanding exact number in the Tsimane’, future research should try to pinpoint what specific factors within the formal education setting are the ones to help number knowledge.

## Supporting information

S1 Appendix US children. (PDF)
