# Supplementary material for: Verbal counting and the timing of number acquisition in an indigenous Amazonian group
Source: PLoS One. 2022 Aug 1;17(8):e0270739. doi: 10.1371/journal.pone.0270739 (PMC9342773; doi:10.1371/journal.pone.0270739)
Supplement: S1 Appendix — (PDF) [file pone.0270739.s001.pdf]

S1 Appendix. US Children.

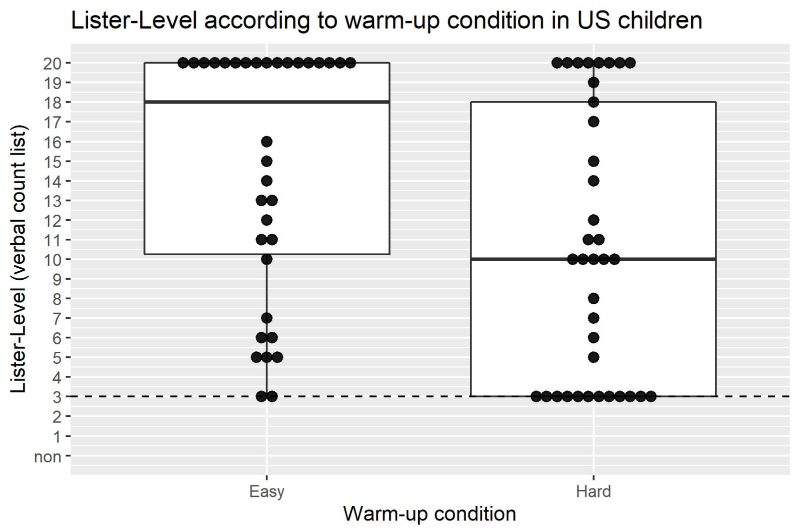

Fig S 1. Lister-level by warm-up in US kids US children's Lister-Level according to the easy (naming animals like "cat" and "dog") or difficult warm-up condition (naming animals like "armadillo" and "platypus")

Recall that warm-up task did not influence Tsimane' children's lister-level performance. (Move-and-Say level and Giver-level weren't affected by warm-up condition, either. Move-and-Say level: $p = 0.14$, $W = 1460.5$, Giver-level: $p = 0.73$, $W = 1299.5$).

As a point of comparison with the Tsimane', we attempted to manipulate willingness to verbalize count list items in US children (n=72, female=28, male=44, ages 2-4 years old) with a warm-up list task. In the warm-up task, we randomly assigned US children to one of two possible conditions. Children were either given an easy (e.g. cat, dog, etc.) or hard (e.g. armadillo, platypus, etc.) set of animals to name. Images of animals were presented on flashcards. Children were given one animal as an example: "This one's a [ANIMAL NAME]" and were subsequently asked to name each new animal with some variant of the phrase: "Can you name this one?". If the child didn't answer for 5 seconds or said they didn't know an answer, a new flashcard was shown.

Next, children were asked to recite the verbal count list two times, via the prompt "Can you count with me? 1, 2, 3....". The first three elements of the count list were presented to each participating child, unless they started listing elements before the

experimenter was able to provide examples. In the latter case, the child was allowed to count until they couldn't provide any further examples (which occurred as soon as 1 or 2 elements in, in some cases). To standardize the comparison between children who listened to the count list examples and children who counted without listening, we grouped any child who named 3 or fewer items into one baseline naming category (for the purposes of analysis and graphical representation). In scoring children's performance, we considered their Lister-Level to be the highest count they reached out of their two attempts, without making any mistakes. Lister-Level was capped at 20.

Fig S 1 illustrates US children's Lister-Levels (y-axis), for the easy and hard warm-up conditions (x-axis). 94% of children across both conditions list above baseline, indicating that children do not appear to be inhibited in listing at least some items of the count list. However, children's naming differs across conditions. The mean number of count list items named by children in the hard condition is 10.51, and in the easy condition is 14.56. This is a significant difference ($p < 0.01$, W=396), based on a comparison of 34 children in the easy condition and 37 children in the hard condition. This differs from our findings in the Tsimane', where children named statistically the same amount of count list items in both warm-up conditions.

Why were we able to affect count list recitation for US children, but not for Tsimane' children? One possibility is that US children's confidence is more tethered to their performance due to US classroom dynamics. Also, different warm-up tasks have different demands. (Asking "Can you name this one?" [image of a cat], for example, is different from asking "Some of my friends are Roberto, Sonia, and Geraldo. Can you name some friends?") We gave different warm-up tasks to US and Tsimane' children in an attempt to calibrate tasks and difficulties to each specific culture. Perhaps we misjudged what types of tasks Tsimane' children would consider "easy" vs. "difficult". Regardless, the warm-up task manipulation appears not to have influenced Tsimane' performance (Fig 2, 3 of main text).
